# Supplementary material for: A Bacterial Microbiome Is Dispensable for the Induction of CD8 T Cell Exhaustion
Source: Eur J Immunol. 2026 Aug 24;56(8):e70238. doi: 10.1002/eji.70238 (PMC13501940; doi:10.1002/eji.70238)
Supplement: Supplementary file 1 [file EJI-56-e70238-s001.pdf]

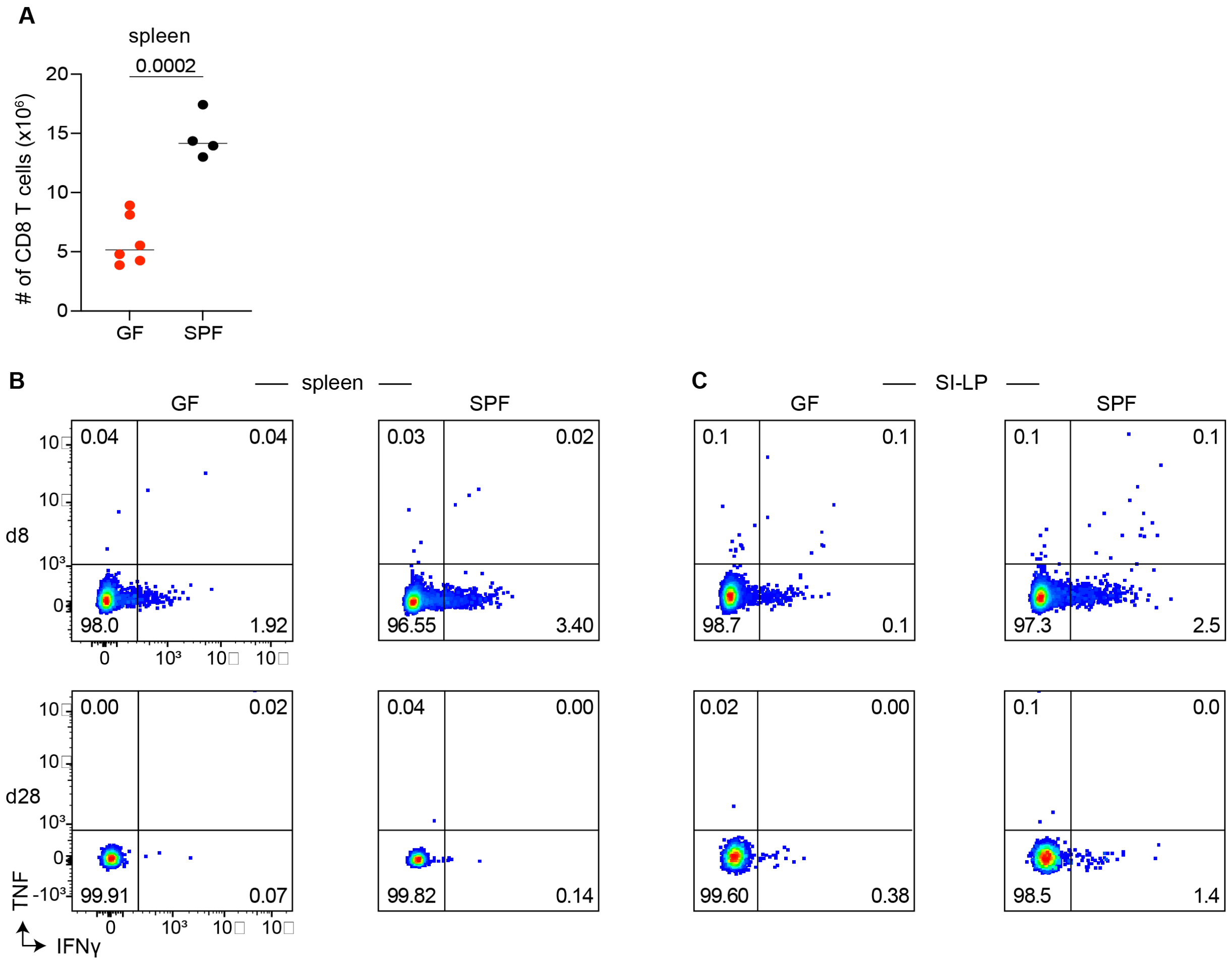

Supp. Fig. 1: CD8 T cell numbers in GF and SPF mice and cytokine Production in CD8 T cells in LCMV infected germ-free and SPF mice. A) Shows the numbers and frequencies of total splenic CD8^{+} T cells in SPF and germ-free mice. **B** and **C**) Experimental design: Germ-free (GF) and SPF C57BL/6 mice were infected with LCMV clone-13 and analyzed on day 8 or d28 after infection. **B** and **C**) Representative, CD8gated flow cytometry plots showing the frequency of IFNγ and TNF expressing cells of GF and SPF mice on day 8 and d28 after infection in the spleen (A) and the SI-LP (B) after *in vitro* incubation in the absence of peptide.

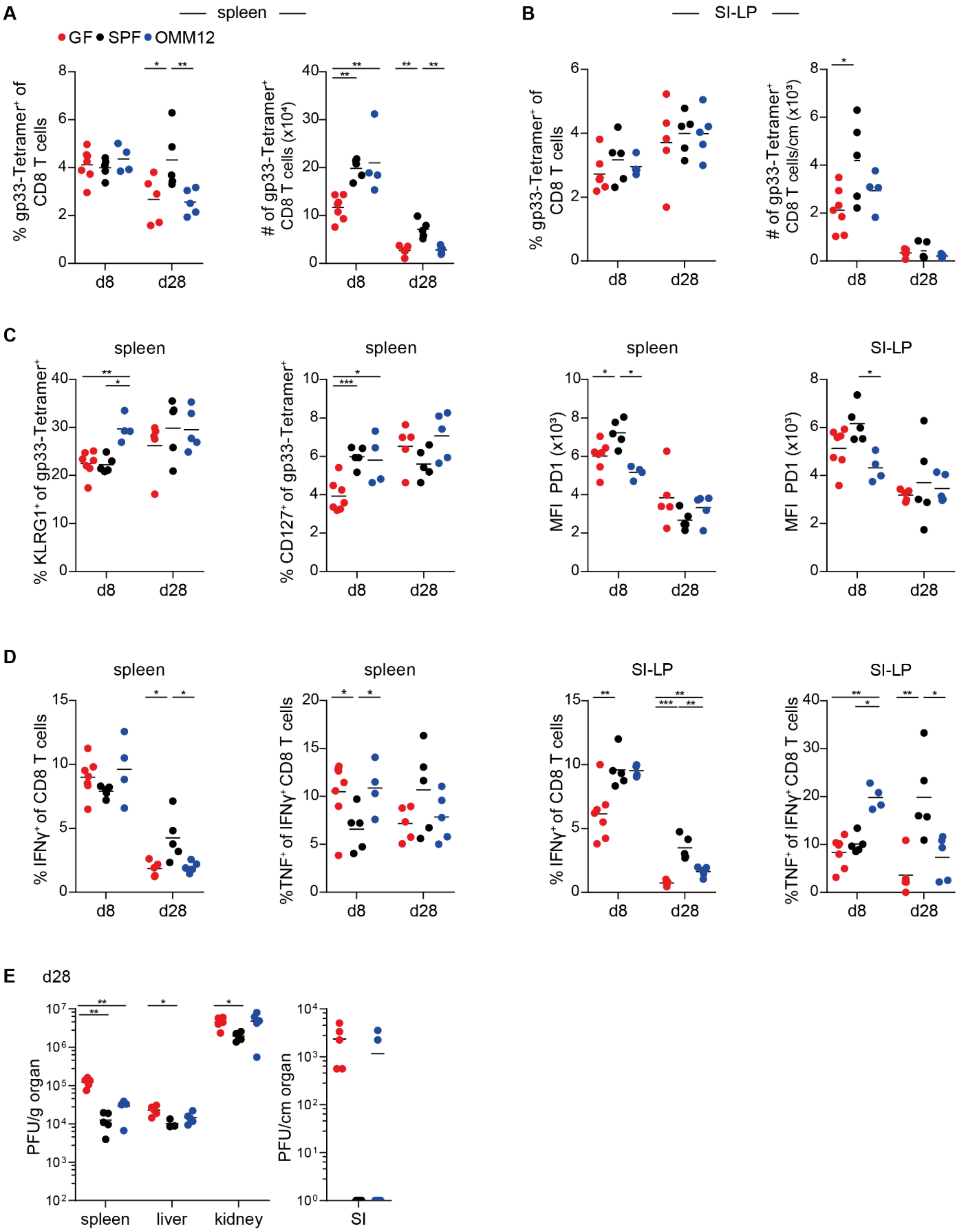

Supp. Fig. 2: Similar phenotypic differentiation of LCMV-specific T cells in germ-free, SPF, and oligoMM-12 mice. Experimental design: Germ-free (GF, red), SPF (black) and oligoMM-

12 recolonized (OMM12, blue) C57BL/6 mice were infected with LCMV clone-13 and analyzed on day 8 or d28 after infection. **A** and **B**) Frequencies (A) and numbers (B) of tetramer$^+$ cells of GF (red), SPF (black) and oligoMM-12 (blue) mice in the spleen and per centimeter of the SI-LP. **C**) Frequencies of KLRG1$^+$ and CD127$^+$ and MFI of PD-1 of tetramer$^+$ cells for GF, SPF and oligoMM-12 mice in the spleen (KLRG1, CD127 and PD-1) and the SI-LP (PD-1). **D**) Frequencies of IFNγ expressing (left) CD8 T cells and frequencies of TNF$^+$ of IFNγ expressing (right) CD8 T cells for GF, SPF, and oligoMM-12 mice. **E**) Viral titers in the spleen, liver, kidney (PFU/g), and SI (PFU/cm) were assessed on day 28 after infection. All data are from a single experiment with at least $n \geq 4$; symbols indicate individual mice; horizontal lines show the mean; P values are from unpaired t-test or Mann-Whitney test (*p value < 0.05, **p value < 0.01, ***p value < 0.001).
